# Supplementary material for: Biophysical comparison of ATP-driven proton pumping mechanisms suggests a kinetic advantage for the rotary process depending on coupling ratio
Source: PLoS One. 2017 Mar 20;12(3):e0173500. doi: 10.1371/journal.pone.0173500 (PMC5358804; doi:10.1371/journal.pone.0173500)
Supplement: S1 File — The analysis presented in the main text compares the kinetics of the rotary and other possible mechanisms coupling ATP hydrolysis to proton transport with an H+:ATP coupling ratio of 3:1. Additional details supporting that analysis are presented here: (1) kinetic diagrams for the possible mechanisms considered, (2) the specific range of conditions used in the analysis, (3) the specific range of parameter values used in the analysis, (4) details of the variations in model assumptions used in the sensitivity analysis, and (5) results from a test of 2:1 H+:ATP coupling ratio. (PDF) [file pone.0173500.s001.pdf]

Supplementary Information for Biophysical comparison of ATP-driven proton pumping mechanisms suggests a kinetic advantage for the rotary process depending on coupling ratio

R. Anandakrishnan and D. M. Zuckerman

December 13, 2016

The analysis presented in the main text compares the kinetics of the rotary and other possible mechanisms coupling ATP hydrolysis to proton transport with an H^+:ATP coupling ratio of 3:1, using the kinetic cycles shown in Fig. S1. Additional details supporting that analysis are presented here: (1) kinetic diagrams for the possible mechanisms considered, (2) the specific range of conditions used in the analysis, (3) the specific range of parameter values used in the analysis, (4) details of the variations in model assumptions used in the sensitivity analysis, and (5) results from a test of 2:1 H^+:ATP coupling ratio.

1 Kinetic diagrams for the mechanisms considered

For a 3:1 H^+:ATP coupling ratio, the H^+ pumping cycle for the rotary, alternating access and other possible mechanisms can be represented by a sequence of ten reactions (events) as shown in Fig. S1. The rate constants (k) for each reaction, shown in the figure, are defined in the *Range of parameter values* section, and the values used are listed in Table S2 below. The difference between the rotary and the basic alternating access mechanisms can be characterized by the order of proton binding and transport. In the rotary mechanism protons bind to the transmembrane V_o subunit of the V-ATPase facing the cytosol (the higer pH/lower proton concentration "cyt" side of the membrane), and are transported across the membrane (to the "out"-side) one-at-a-time [1]. An alternating access mechanism on the other hand can bind multiple protons to the high pH cytosol side of the ATPase and simultaneously transport all of them across the membrane [2]. Results for all five possible proton transport orderings for the 3:1 H^+:ATP coupling ratio shown in Fig. S1, are presented in the main text. The sensitivity analysis described in section 5 below also presents the results for additional non-ion event orders (i.e., the order of ATP, ADP, and Pi binding/unbinding and ATP hydrolysis/synthesis).

2 Thermodynamic cycle constraint

The parameters (rate constants) for the steps in the kinetic cycles shown in Fig. S1 are constrained by the thermodynamic requirement that the free energy change for the proton pumping cycle, which is the

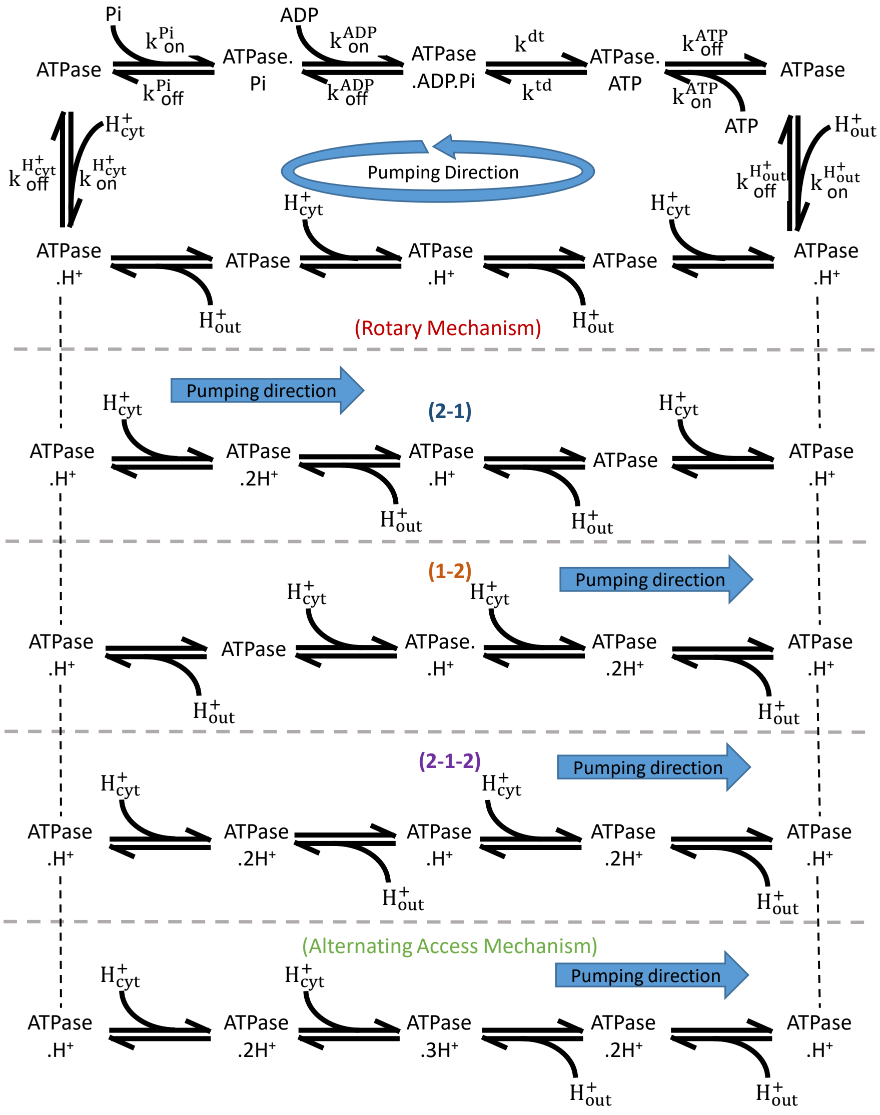

Figure S1: Minimalist kinetic models of rotary and alternative mechanisms for proton pumps with a 3:1 H^+:ATP coupling ratio. The parameters (rate constants) in the kinetic models implicitly include the effect of conformational changes in a thermodynamically consistent manner.

driving potential ΔG_{driv}, be equal to the sum of the free energy changes for each of the steps in the

cycle [3], i.e.

$$\Delta G_{\rm driv} = \sum_{i=1}^{s} G_i - G_{i+1} \tag{1}$$

where the basic free energy (FE) G_i for state i is defined in terms of the effective first order rate constants $\alpha_{i,j}$ by

$$G_{i-1} - G_i = -(1/\beta)\ln(\alpha_{i,i-1}/\alpha_{i-1,i}) \tag{2}$$

where $\alpha_{i,j} = c_i k_{i,j}$ is the effective first order rate constant, with $c = 1$ for a first order process and $c = [X]$ when species X binds in the transition from state i to j, and $k_{i,j}$ is the corresponding rate constant; $\beta = 1/k_B T$ where k_B is the Boltzmann constant and T is the temperature. $\Delta G_{\rm driv}$ is also defined as

$$\Delta G_{\rm driv} = \Delta G_{\rm ATP} - n(-F\Delta\psi + 2.3RT\Delta{\rm pH}) \tag{3}$$
$$\Delta G_{\rm ATP} = \Delta G^0 + RT\ln([ATP]/[ADP][Pi]) \tag{4}$$
$$10^{\Delta{\rm pH}} = [H^+_{\rm cyt}]/[H^+_{\rm out}] \tag{5}$$

where n is the coupling ratio, $\Delta\psi = \psi_{\rm cyt} - \psi_{\rm out}$ is the trans-membrane potential and $\Delta{\rm pH} = {\rm pH}_{\rm cyt} - {\rm pH}_{\rm out}$ is the pH difference (the "cyt"-side is the higher pH catalytic side of the membrane and the "out"-side is the lower pH side of the membrane [4]); $\Delta G_{\rm ATP}$ is the free energy required for ATP hydrolysis for a given concentrations of ADP, ATP and Pi under ideal-solution assumptions; ΔG^0 is the free energy required for ATP hydrolysis under equilibrium concentrations of ADP, ATP and Pi. Since ΔG^0 depends on pH [5], we use different values for ΔG^0 depending on pH. See Sensitivity Analysis below. F is the Faraday constant and R is the gas constant. The ratio $\alpha_{i,i+1}/\alpha_{i+1,i} = c_i k_{i,i+1}/c_{i+1}k_{i+1,i} = c_i K/c_{i+1}$ where K is the equilibrium constant for the step from state i to $i+1$. From Eqs. (1)–(5), along with this relationship between the ratio of effective first order rate constants $\alpha_{i,j}$ and the equilibrium constant K, we have

$$e^{n\beta F\Delta\psi} = \frac{K_d^{ATP}}{K_d^{ADP}K_d^{Pi}K^{dt}}\left(\frac{K_d^{H^+_{\rm out}}}{K_d^{H^+_{\rm cyt}}}\right)^n e^{-\beta\Delta G^0} \tag{6}$$

Eq. (6) is similar to one previously suggested by Boork and Wennerström [6]. This constraint applies to all five mechanisms considered. Note that the constraint depends only on the equilibrium constants, ΔpH and $\Delta\psi$. We chose to set the rate constant $k_{\rm off}^{H^+_{\rm out}}$ using the above thermodynamic constraint in our model, while using the optimization protocol described in the main text for setting all other rate constants, i.e. from Eq. (6),

$$k_{\rm off}^{H^+_{\rm out}} = \left(K_d^{ADP}K_d^{Pi}K^{dt}/K_d^{ATP}\right)^{1/n} K_d^{H^+_{\rm cyt}} k_{\rm on}^{H^+_{\rm out}} e^{\beta\Delta G^0} e^{\beta F\Delta\psi} \tag{7}$$

Since the optimization protocol determines the set of parameters that maximize the rate H^+/s, the resulting set of optimized parameter values do not depend on which specific parameter is selected as the dependent parameter in the thermodynamic constraint, Eq. (7).

3 Range of conditions

Rates of H^+/s were calculated for a range of possible conditions, based on values reported in the literature [4, 7, 8, 9, 10]. These ranges were extended beyond the reported ranges to represent potentially

evolutionary and pathological states that may be more extreme than typical physiological conditions (Table S1). Note that pH_{out} represents the extracellular pH value for plasma membrane ATPase and the organelle pH value for organelle membrane ATPase. The proton pump transports $H^+$from the cytosol side of the membrane to the "out" side, with transmembrane $\Delta pH = pH_{cyt} - pH_{out}$ and $\Delta\psi = \psi_{cyt} - \psi_{out}$. For the parameter optimization protocol described in *Methods* in the main text, and for the test results shown in the main text and in here, conditions were randomly sampled from the the range of values shown in Table S1, with the exception of ΔG_{driv} and ΔG^0 (Eq. (3)). To limit the range of values to conditions consistent with proton pumping, values that resulted in a negative driving potential were excluded. The concentration values for ADP and Pi were sampled from a uniform distribution in log space and all other values were sampled in linear space.

Table S1: Range of conditions. Ranges based on reported physiological conditions for plant cell plasma and vacuolar membranes [4, 7, 8, 9, 10]. Also listed are the condition values used for Fig. 5 of the main text. Note that pH_{out}represents the extracellular pH value for plasma membrane ATPase and the organelle pH for organelle membrane ATPase, with $\Delta pH = pH_{cyt} - pH_{out}$, and $\Delta\psi = \psi_{cyt} - \psi_{out}$.

Condition	Literature	Range used	Values for Fig. 5 of main text
pH_{cyt}	7.2 – 7.5 [7]	6.5–8.5	7.5
[ATP]/[ADP]	17 [9]	10 – 30	20
ΔpH	1 – 6 [7]	0 – 3	1
$\Delta\psi$(mV)	-30 – -160 [4]	0 – -250	-80
[ADP] (M)	3×10^{-5} [9]	3×10^{-4} – 8×10^{-6}	3×10^{-5}
[Pi] (mM)	10^{-2} – 10^{-3} [10]	10^{-2} – 10^{-3}	$10^{-2.5}$
ΔG_{driv} (kcal/mol)	0 – 14	0 – 14	3.1
ΔG^0 (kcal/mol)	7.6 [10]	7.6	7.6

4 Range of parameter values

The range of realistic parameter values are limited by physical and structural constraints [11, 12]. The parameter values reported in the literature vary considerably [13, 14, 15, 16, 17, 18, 19]. For the purpose of this analysis we use an optimization range spanning one order of magnitude around a representative set of values chosen from the literature, as shown in Table S2. Each step in the process shown in Fig. S1 has a binding k_{on} (or forward k_{td} in the case of the ATP hydrolysis step) and an unbinding k_{off} (or k_{dt} for the phosphorylation of ADP) rate constant. $K_d = k_{off}/k_{on}$ and $K^{dt} = k_{dt}/k_{td}$ are the dissociation constants and the equilibrium constant for ADP to ATP phosphorylation, respectively. We also defined a cooperativity factor β, which represents the cooperativity between the proton binding sites, i.e. the effect of the (un)binding of one H^+ on the binding affinity of a subsequent H^+. Specifically, $K_d^{H^+_{out2}} = K_d^{H^+_{out1}} 10^{\beta_{out1}}$, $K_d^{H^+_{out3}} = K_d^{H^+_{out2}} 10^{\beta_{out2}}$, $K_d^{H^+_{cyt2}} = K_d^{H^+_{cyt1}} 10^{\beta_{cyt1}}$, and $K_d^{H^+_{cyt3}} = K_d^{H^+_{cyt2}} 10^{\beta_{cyt2}}$.

5 Sensitivity analysis

The results from extensive and systematic sensitivity analysis presented here, show that our main results are qualitatively insensitive to model assumptions (Table S3, Fig. S2 – S10). The model assumptions

Table S2: Parameter ranges for optimization. k_{on} are the binding rate constants, K_d the dissociation constants, k_{dt} and K^{dt} the ADP phosphorylation rate and equilibrium constants, and β represents the cooperativity factor between consecutive H^+binding and transport as described in the text. Also listed are the parameter values used for Fig. 5 of the main text. The thermodynamic cycle constraint, described above, is used to calculate the value for $K_{\text{off}}^{H^+_{\text{out}}}$.

Parameter	Literature	Value/Range used	Values for Fig. 5 of main text
k_{on}^{Pi} (M^{-1} s^{-1})	12 [14]	$1 - 10^2$	10^2
k_{on}^{ADP} (M^{-1} s^{-1})	4.2×10^6 [14]	$4 \times 10^5 - 4 \times 10^7$	4×10^7
k_{on}^{ATP} (M^{-1} s^{-1})	4.0×10^7 [14]	$4 \times 10^6 - 4 \times 10^8$	4×10^8
$k_{\text{on}}^{H^+_{\text{out}}}$ (M^{-1} s^{-1})	4×10^{10} [13]	$4 \times 10^9 - 4 \times 10^{11}$	4×10^{11}
$k_{\text{on}}^{H^+_{\text{cyt}}}$ (M^{-1} s^{-1})	4×10^{10} [13]	$4 \times 10^9 - 4 \times 10^{11}$	4×10^{11}
K_d^{Pi} (M)	17 [14]	$2 - 2 \times 10^2$	2
K_d^{ADP} (M)	8.6×10^{-5} [14]	$9 \times 10^{-4} - 9 \times 10^{-6}$	9×10^{-6}
K_d^{ATP} (M)	4.9×10^{-4} [14]	$5 \times 10^{-3} - 5 \times 10^{-5}$	5×10^{-3}
$K_d^{H^+_{\text{out}}}$ (M)	1.6×10^{-6} [18]	Thermodynamic Constraint	
$K_d^{H^+_{\text{cyt}}}$ (M)	1.6×10^{-8} [18]	$2 \times 10^{-7} - 2 \times 10^{-9}$	2×10^{-7}
k_{dt} (s^{-1})	120 [14]	$10 - 10^3$	10^3
K^{dt}	0.9 [14]	0.1 - 10	0.1
$10^{\beta_{\text{out1}}}$		$10^{-1} - 10^{+1}$	10^{+1}
$10^{\beta_{\text{cyt1}}}$		$10^{-1} - 10^{+1}$	10^{+1}
$10^{\beta_{\text{out2}}}$		$10^{-1} - 10^{+1}$	10^{-1}
$10^{\beta_{\text{cyt2}}}$		$10^{-1} - 10^{+1}$	10^{-1}

include event order (reaction sequence), optimization protocol for kinetic parameters, optimized parameter values, and the range of values for pH on the high pH side of the membrane, pH_{cyt}. The baseline (default) set of model assumptions are shown in the first row of data in Table S3, and the corresponding results in Fig. S2. All results are based on five sets of >30000 random conditions sampled from the range of values shown in Table S1 as described in the *Range of conditions* section above.

In addition to the baseline event order (Fig. S1), tests of five other event orders (model assumptions 2 – 6 in Table S3) show that the results are qualitatively insensitive to event order (Fig. S3 and S4). In the table, "Free" refers to the free ATPase, ".ATP" refers to ATP bound state, ".ADP.Pi" refers to ADP and Pi bound state after ATP hydrolysis, ".ADP" refers to the ADP bound state after Pi release, ".Pi" refers to the Pi bound state after ADP release, and "$3\times H^+$" refers to the transport of three protons in the order shown in Fig. S1 for the different mechanisms.

The evolution-inspired optimization protocol separately optimizes the parameters for each mechanism, *without fitting*, to maximize the rate of proton transport (H^+/s) under challenging conditions [20]. See Methods in the main text. The baseline optimization protocol selects the lower 10 percentile of parameter sets (models) characterizing the "challenging conditions" (Fig. S2). Tests of three additional characterizations of challenging conditions, the lower 0 percentile, 20 percentile and 50 percentile, show that the results are qualitatively insensitive to event order (Fig. S5 and S6).

In addition to the parameters determined by the optimization protocol, we tested four different

Table S3: Summary data from sensitivity analysis. Performance of possible alternative mechanisms relative to the rotary mechanism for different model assumptions. Average ratio is geometric average of H^+/s relative to rotary mechanism. Model assumption 1 represents the baseline (default) model corresponding to results shown in Fig. S2. The Event Orders and Parameter Ranges are described in the text.

Model assumptions					Average H^+/s ratio			
Id	Event order	Opt. protocol	Parameter ranges/values	pH_{cyt} ranges	$\frac{2-1}{\text{rotary}}$	$\frac{1-2}{\text{rotary}}$	$\frac{2-1-2}{\text{rotary}}$	$\frac{\text{alt acc}}{\text{rotary}}$
1	- Free - .ATP - .ADP.Pi - .ADP - Free - 3×H^+- (Fig. S1)	10%	From literature (Table S2)	6.5–8.5	0.54	0.65	0.48	0.23
2	- Free - .ATP - .ADP.Pi - 3×H^+- .Pi - Free -	—"—	—"—	—"—	0.52	0.72	0.49	0.20
3	- Free - .ATP - .ADP.Pi - 3×H^+- .ADP - Free -	—"—	—"—	—"—	0.52	0.74	0.59	0.22
4	- Free - .ATP - .ADP.Pi - .ADP - 3×H^+- Free -	—"—	—"—	—"—	0.75	0.69	0.61	0.31
5	- Free - .ATP - .ADP.Pi - .Pi - 3×H^+- Free -	—"—	—"—	—"—	0.56	0.79	0.46	0.25
6	- Free - .ATP - .ADP.Pi - .Pi - 3×H^+- Free -	—"—	—"—	—"—	0.73	0.74	0.59	0.32
7	- Free - .ATP - .ADP.Pi - .ADP - Free - 3×H^+- (Fig. S1)	0%	From literature (Table S2)	6.5–8.5	0.28	0.65	0.25	0.08
8	—"—	20%	—"—	—"—	0.52	0.62	0.46	0.20
9	—"—	50%	—"—	—"—	0.55	0.59	0.46	0.19
10	- Free - .ATP - .ADP.Pi - .ADP - Free - 3×H^+- (Fig. S1)	10%	Random set 1	6.5–8.5	0.73	0.72	0.61	0.37
11	—"—	—"—	Random set 2	—"—	0.55	0.81	0.50	0.38
12	—"—	—"—	Random set 3	—"—	0.56	0.56	0.41	0.20
13	—"—	—"—	Random set 4	—"—	0.77	0.73	0.69	0.40
14	- Free - .ATP - .ADP.Pi - .ADP - Free - 3×H^+- (Fig. S1)	10%	From literature (Table S2)	5.5–7.5	0.80	0.94	0.80	0.03
15	—"—	—"—	—"—	7.5–8.5	0.30	0.52	0.29	0.15

Table S4: Random set of parameter values used for sensitivity analysis.

Parameter	Random set 1	Random set 2	Random set 3	Random set4
k_{on}^{Pi} (M^{-1} s^{-1})	$10^{0.61}$	$10^{0.87}$	$10^{1.02}$	$10^{1.43}$
k_{on}^{ADP} (M^{-1} s^{-1})	$4 \times 10^{5.29}$	$4 \times 10^{5.84}$	$4 \times 10^{6.79}$	$4 \times 10^{6.40}$
k_{on}^{ATP} (M^{-1} s^{-1})	$4 \times 10^{6.19}$	$4 \times 10^{6.66}$	$4 \times 10^{7.79}$	$4 \times 10^{6.43}$
$k_{\text{on}}^{H^+_{\text{out}}}$ (M^{-1} s^{-1})	$4 \times 10^{9.37}$	$4 \times 10^{9.41}$	$4 \times 10^{9.25}$	$4 \times 10^{10.95}$
$k_{\text{on}}^{H^+_{\text{cyt}}}$ (M^{-1} s^{-1})	$4 \times 10^{9.37}$	$4 \times 10^{9.41}$	$4 \times 10^{9.25}$	$4 \times 10^{10.95}$
K_d^{Pi} (M)	$2 \times 10^{0.83}$	$2 \times 10^{0.87}$	$2 \times 10^{1.10}$	$2 \times 10^{1.93}$
K_d^{ADP} (M)	$9 \times 10^{-5.44}$	$9 \times 10^{-4.05}$	$9 \times 10^{-5.42}$	$9 \times 10^{-5.10}$
K_d^{ATP} (M)	$5 \times 10^{-3.00}$	$5 \times 10^{-4.10}$	$5 \times 10^{-3.58}$	$5 \times 10^{-4.95}$
$K_d^{H^+_{\text{cyt}}}$ (M)	$2 \times 10^{-7.69}$	$2 \times 10^{-8.24}$	$2 \times 10^{-7.42}$	$2 \times 10^{-7.01}$
k_{dt} (s^{-1})	119.57	34.18	76.14	74.06
K^{dt}	0.62	0.40	0.13	0.32

randomly generated sets of parameter values. The parameters values were sampled from a uniform distribution in log space in the vicinity (±1 order of magnitude) of the literature values (Table S2). The results were qualitatively insensitive to parameter values (Fig. S7 and S8), The specific set of randomly generated parameter values are listed in Table S4.

We also considered three pH$_{\text{cyt}}$ ranges: 5.5–7.5, 6.5–8.5 and 7.5–9.5 showing that the results are qualitatively insensitive to pH$_{\text{cyt}}$ range (Fig. S9 and S10). Since the FE of ATP phosphorylation ΔG^0 depends on pH [5], we use different values for the three different ranges approximately representing the average value for ΔG^0 within the range of pH values: 6.7 kcal/mol for 5.5–7.5, 7.6 kcal/mol for 6.5–8.5, and 8.3 kcal/mol for 7.5–9.5.

Each set of model assumption was tested for the full range of conditions listed in Table S1 and the results were qualitatively similar. Table S3 and Fig. S2–S10 show that the results are qualitatively insensitive to model assumptions, with the rotary mechanism, on average, being faster than other possible mechanisms across a wide range of conditions. These results are summarized in Fig. 4(c) of the main text.

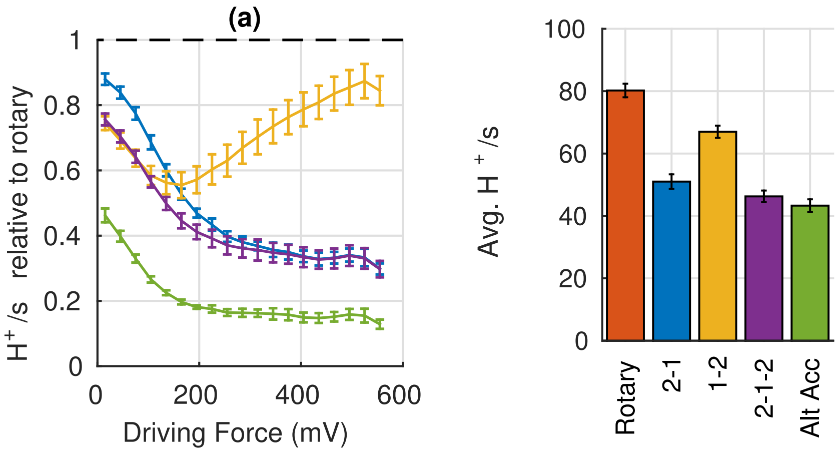

Figure S2: Results for baseline (default) model assumptions (3:1 H^+:ATP coupling ratio). (a) Geometric average ratio of H^+/s relative to rotary mechanism. (b) Average rate of H^+/s. Baseline assumptions are shown in Table S3, model assumption 1. Error bars show the standard error of the mean when sampling over a range of conditions. Connecting lines are shown to guide the eye.

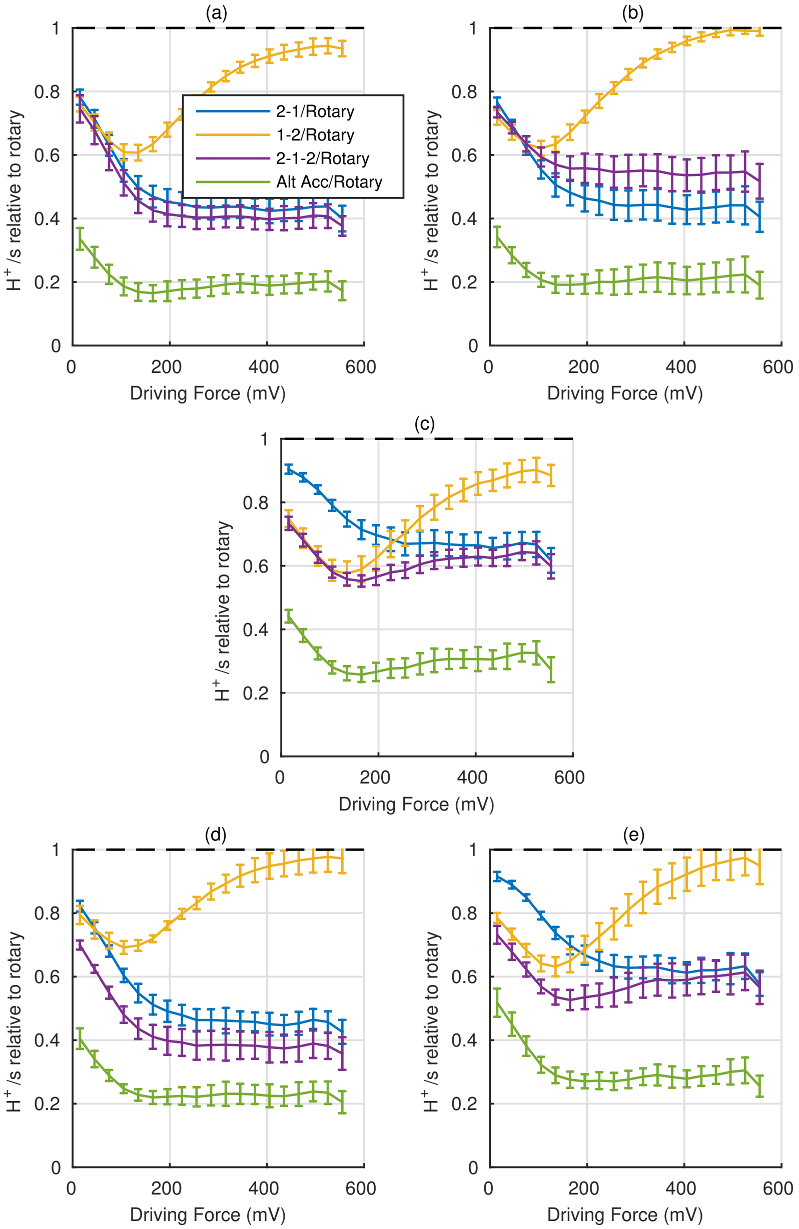

Figure S3: Sensitivity of average ratio to event order (3:1 H^+:ATP coupling ratio). (a) – (e) show the geometric average ratio relative to the rotary mechanism, for event orders corresponding to model assumption 2 – 6 in Table S3. Results are qualitatively similar to the results for the baseline event order shown in Fig. S2. Error bars show the standard error of the mean when sampling over a range of conditions. Connecting lines are shown to guide the eye.

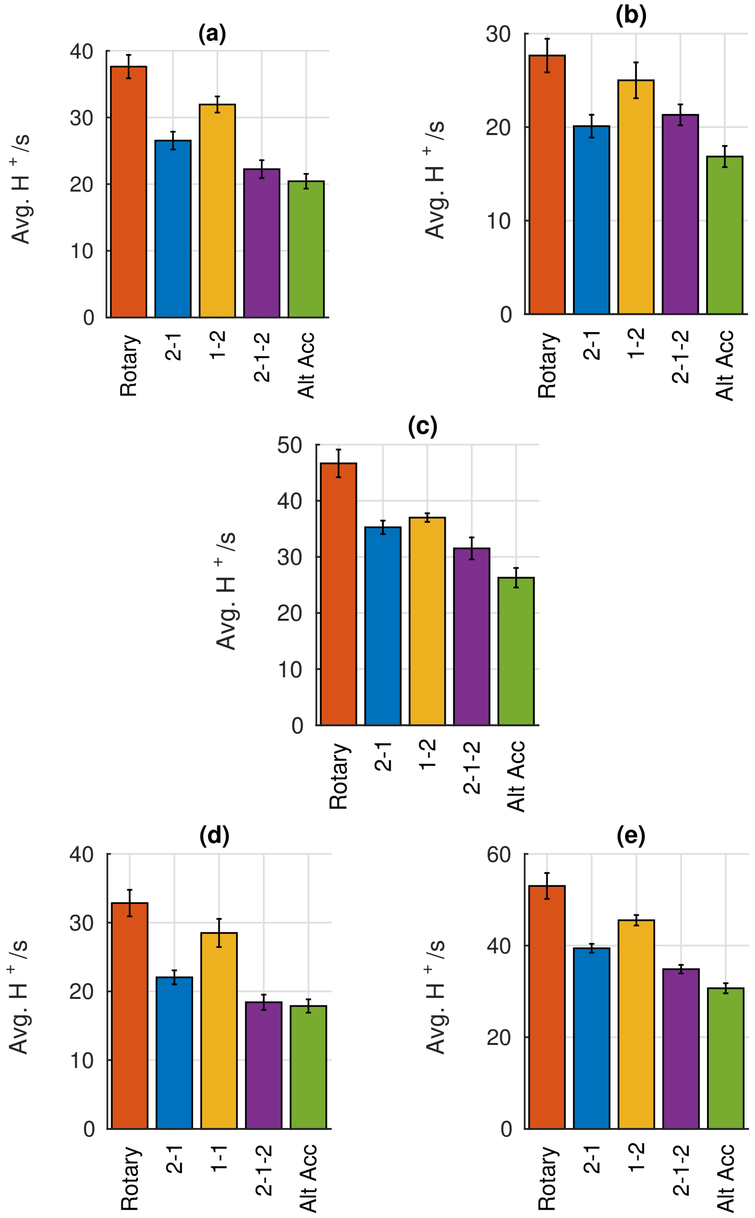

Figure S4: Sensitivity of rate of H^+/s to event order (3:1 H^+:ATP coupling ratio). (a) – (e) show the average H^+/s for event orders corresponding to model assumption 2 – 6 in Table S3. Results are qualitatively similar to the results for the baseline event order shown in Fig. S2. Error bars show the standard error of the mean when sampling over a range of conditions.

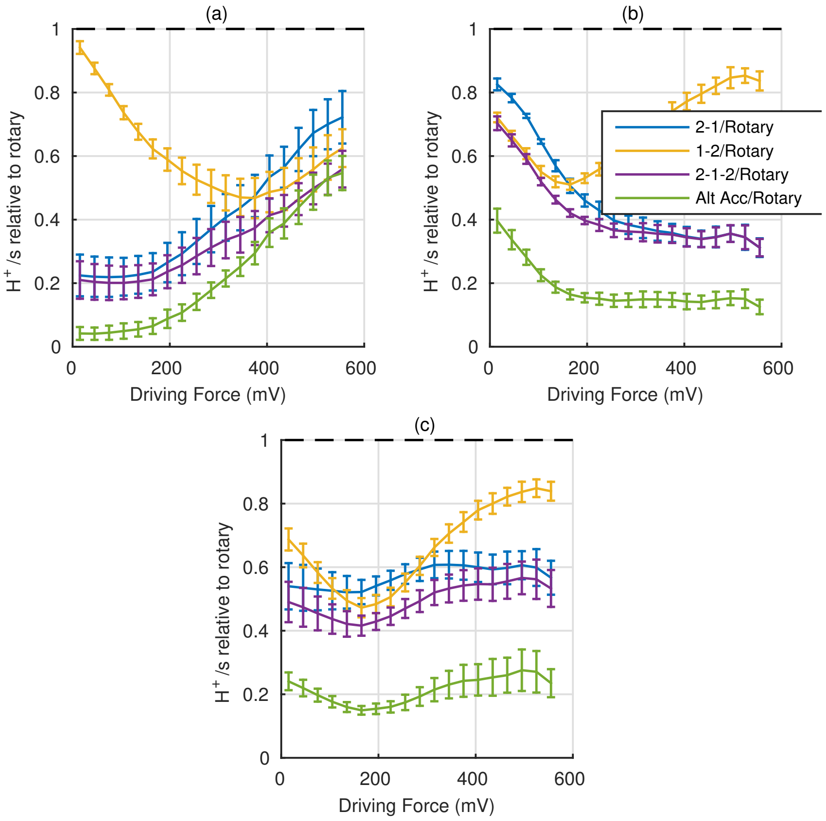

Figure S5: Sensitivity of average ratio to optimization protocol (3:1 H^+:ATP coupling ratio). Geometric average ratio relative to the rotary mechanism for the lower (a) 0 percentile, (b) 20 percentile, and (c) 50 percentile of parameter sets characterizing the challenging condition, corresponding to model assumptions $7-9$ in Table S3. Results are qualitatively similar to the results for the baseline optimization protocol shown in Fig. S2. Error bars show the standard error of the mean when sampling over a range of conditions. Connecting lines are shown to guide the eye.

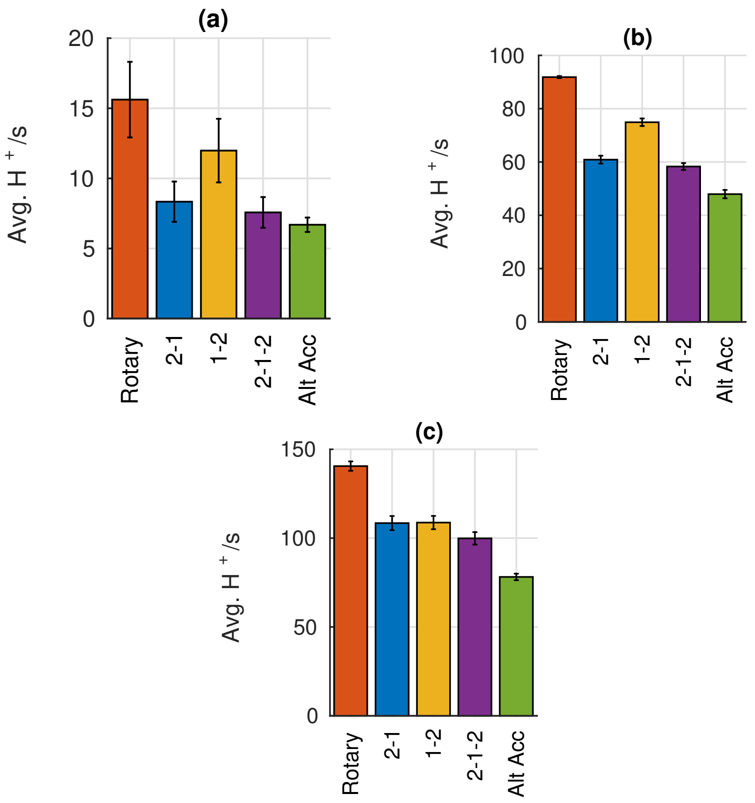

Figure S6: Sensitivity of H^+/s to optimization protocol (3:1 H^+:ATP coupling ratio). Average H^+/s for the lower (a) 0 percentile, (b) 20 percentile, and (c) 50 percentile of parameter sets characterizing the challenging condition, corresponding to model assumptions 7 – 9 in Table S3. Results are qualitatively similar to the results for the baseline optimization protocol shown in Fig. S2. Error bars show the standard error of the mean when sampling over a range of conditions.

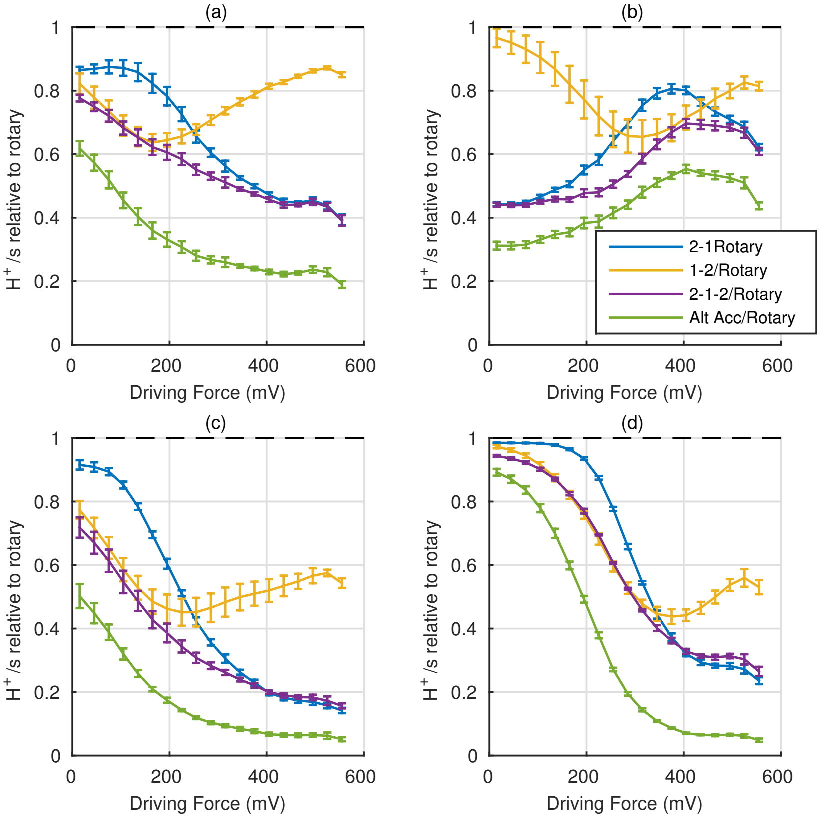

Figure S7: Sensitivity of average ratio to parameter values (3:1 H^+:ATP coupling ratio). (a) – (d) show the geometric average ratio relative to the rotary mechanism, for random sets of parameter values corresponding to random sets 1 – 4 in Table S4. Results are qualitatively similar to the results for the baseline parameter values shown in Fig. S2. Error bars show the standard error of the mean when sampling over a range of conditions. Connecting lines are shown to guide the eye.

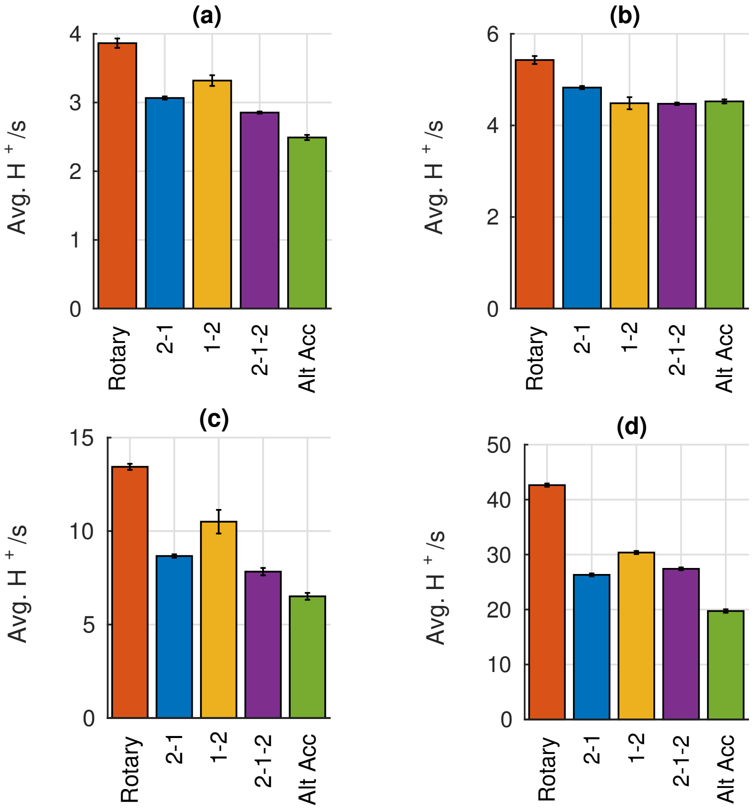

Figure S8: Sensitivity of H^+/s to parameter values (3:1 H^+:ATP coupling ratio). (a) – (d) show the average rate for random sets of parameter values corresponding to random sets $1-4$ in Table S4. Results are qualitatively similar to the results for the baseline parameter values shown in Fig. S2. Error bars show the standard error of the mean when sampling over a range of conditions.

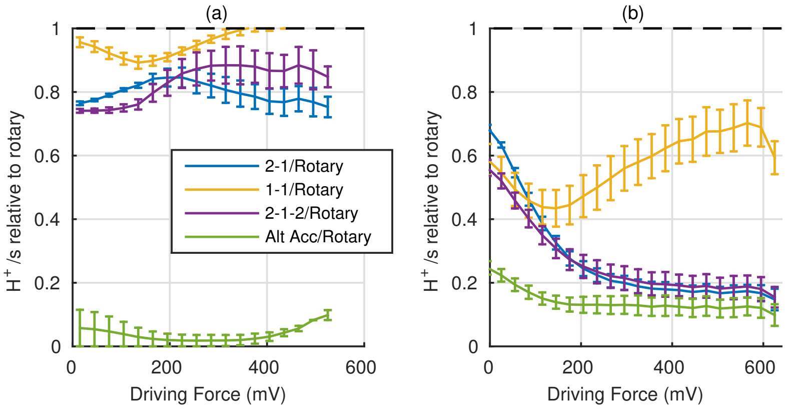

Figure S9: Sensitivity of average ratio to pH_{cyt} (3:1 H^+:ATP coupling ratio). Geometric average ratio relative to the rotary mechanism for the pH_{cyt} range of (a) 5.5 – 7.5 and (b) 7.5 – 9.5, corresponding to model assumptions 14 and 15 in Table S3, respectively. Results are qualitatively similar to the results for the baseline pH range shown in Fig. S2. Error bars show the standard error of the mean when sampling over a range of conditions. Connecting lines are shown to guide the eye.

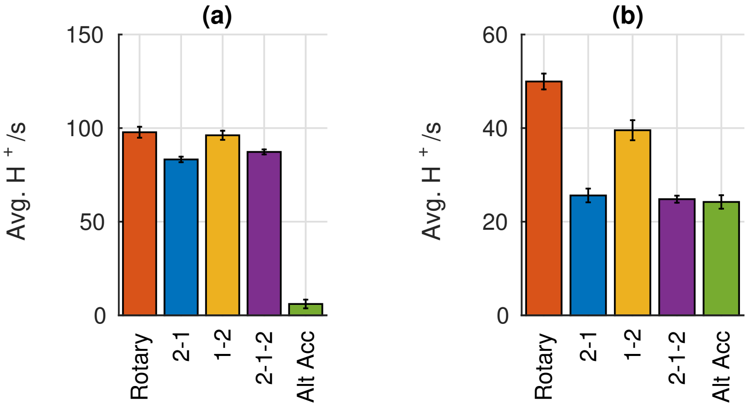

Figure S10: Sensitivity of H^+/s to pH_{cyt} (3:1 H^+:ATP coupling ratio). Average H^+/s for the pH_{cyt} range of (a) 5.5 – 7.5 and (b) 7.5 – 9.5, corresponding to model assumptions 14 and 15 in Table S3, respectively. Results are qualitatively similar to the results for the baseline pH range shown in Fig. S2. Error bars show the standard error of the mean when sampling over a range of conditions.

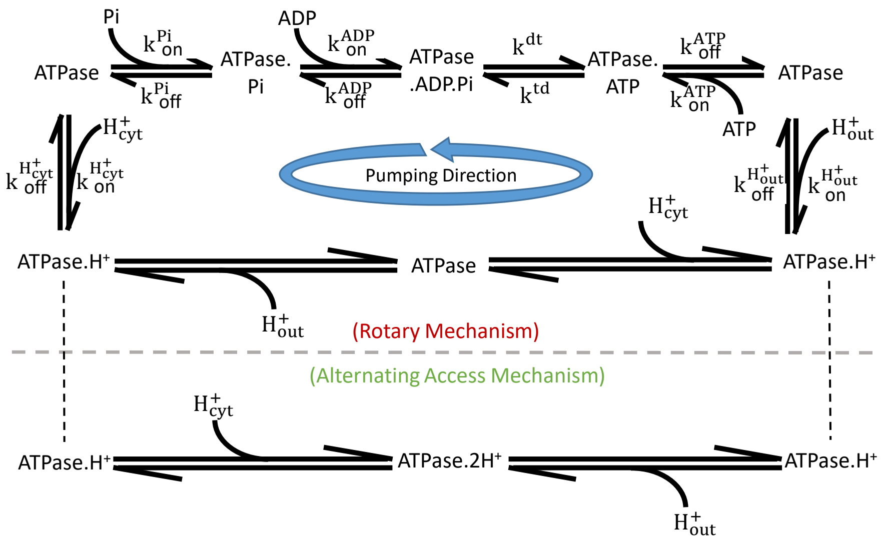

Figure S11: Minimalist kinetic modeling of rotary and alternative mechanisms for proton pumps with 2:1 coupling ratio. Note that the kinetic parameters implicitly include conformational transitions in the binding and chemical processes shown in a thermodynamically consistent way.

6 2:1 H^+:ATP coupling ratio

For the preceding results we chose a 3:1 H^+:ATP coupling ratio, approximately representative of the extensively studied *S. cerevisiae* (yeast) V-ATPase with a coupling ratio of 10:3 [2]. Other V-ATPases use lower coupling ratios, e.g. 2:1 for plant vacuolar V-ATPase [4]. A 2:1 coupling ratio permits only two possible H^+ transport orders corresponding to the rotary one-at-a-time proton transport and the alternating access all-at-once proton transport, as shown in Fig. S11. Fig. S12 shows that the results for a 2:1 coupling ratio are qualitatively similar to the results for the 3:1 coupling ratio (Fig. S2). The same range of conditions and parameters shown in Tables S1 and S2 were used here.

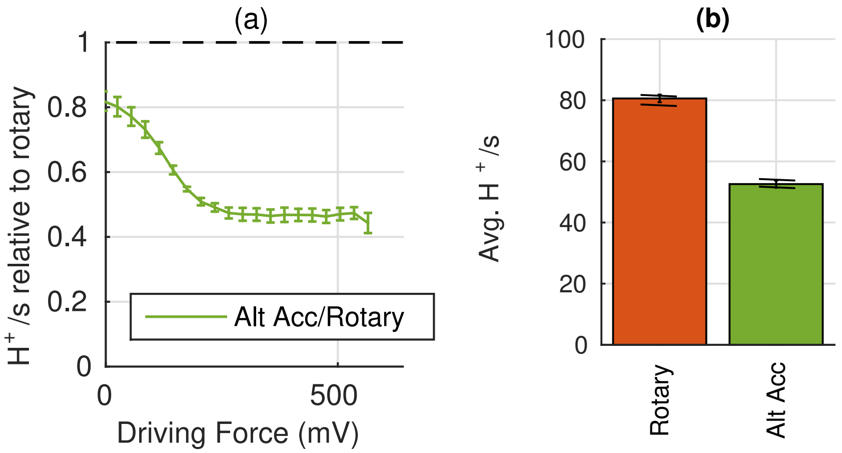

Figure S12: Test of 2:1 H^+:ATP coupling ratio. (a) Geometric average ratio of H^+/s relative to rotary mechanism. (b) Average rate of proton transport. Results are qualitatively similar to the results for the 3:1 coupling ratio shown in Fig. S2. Error bars show the standard error of the mean when sampling over a range of conditions. Connecting lines are shown to guide the eye.

References

References

[1] Werner Kühlbrandt and Karen M. Davies. Rotary ATPases: A New Twist to an Ancient Machine. *Trends in Biochemical Sciences*, 41(1):106–116, January 2016.

[2] Jianhua Zhao, Samir Benlekbir, and John L. Rubinstein. Electron cryomicroscopy observation of rotational states in a eukaryotic V-ATPase. *Nature*, 521(7551):241–245, May 2015.

[3] Terrell L. Hill. *Free Energy Transduction and Biochemical Cycle Kinetics.* Dover Publications, December 2004.

[4] Heven Sze, Xuhang Li, and Michael G. Palmgren. Energization of Plant Cell Membranes by H+-Pumping ATPases: Regulation and Biosynthesis. *Plant Cell*, 11(4):677–689, April 1999.

[5] Robert A. Alberty. Effect of pH and Metal Ion Concentration on the Equilibrium Hydrolysis of Adenosine Triphosphate to Adenosine Diphosphate. *J. Biol. Chem.*, 243(7):1337–1343, April 1968.

[6] Jeff Boork and Håkan Wennerström. The influence of membrane potentials on reaction rates. Control in free-energy-transducing systems. *Biochim. Biophys. Acta, Bioenerg.*, 767(2):314–320, November 1984.

[7] Jon K. Pittman. Multiple Transport Pathways for Mediating Intracellular pH Homeostasis: The Contribution of H(+)/ion Exchangers. *Front. Plant Sci.*, 3(11):1–8, 2012.

[8] Bjorn P. Pedersen, Morten J. Buch-Pedersen, J. Preben Morth, Michael G. Palmgren, and Poul Nissen. Crystal structure of the plasma membrane proton pump. *Nature*, 450(7172):1111–1114, December 2007.

[9] Elisabeth Gout, Fabrice Rébeillé, Roland Douce, and Richard Bligny. Interplay of Mg2+, ADP, and ATP in the cytosol and mitochondria: unravelling the role of Mg2+ in cell respiration. *Proc. Natl. Acad. Sci. U.S.A.*, 111(43):E4560–E4567, October 2014.

[10] Todd P. Silverstein. An exploration of how the thermodynamic efficiency of bioenergetic membrane systems varies with c-subunit stoichiometry of F1F0 ATP synthases. *J. Bioenerg. Biomemb.*, 46(3):229–241, 2014.

[11] Magnus Johansson, Martin Lovmar, and Måns Ehrenberg. Rate and accuracy of bacterial protein synthesis revisited. *Curr. Opin. Microbiol.*, 11(2):141–147, April 2008.

[12] Athel Cornish-Bowden. *The Pursuit of Perfection: Aspects of Biochemical Evolution.* Oxford University Press, 1st edition, November 2004.

[13] O. A. Gopta, D. A. Cherepanov, W. Junge, and A. Y. Mulkidjanian. Proton transfer from the bulk to the bound ubiquinone Q(B) of the reaction center in chromatophores of Rhodobacter sphaeroides: retarded conveyance by neutral water. *Proc. Natl. Acad. Sci. U.S.A.*, 96(23):13159–13164, November 1999.

[14] Joanne A. Baylis Scanlon, Marwan K. Al-Shawi, Nga P. Le, and Robert K. Nakamoto. Determination of the Partial Reactions of Rotational Catalysis in F1-ATPase. *Biochem.*, 46(30):8785–8797, July 2007.

[15] Kengo Adachi, Kazuhiro Oiwa, Takayuki Nishizaka, Shou Furuike, Hiroyuki Noji, Hiroyasu Itoh, Masasuke Yoshida, and Kazuhiko Kinosita. Coupling of Rotation and Catalysis in F1-ATPase Revealed by Single-Molecule Imaging and Manipulation. *Cell*, 130(2):309–321, July 2007.

[16] Kengo Adachi, Kazuhiro Oiwa, Masasuke Yoshida, Takayuki Nishizaka, and Kazuhiko Kinosita. Controlled rotation of the F1-ATPase reveals differential and continuous binding changes for ATP synthesis. *Nat. Commun.*, 3:1022+, August 2012.

[17] C. Grubmeyer, R. L. Cross, and H. S. Penefsky. Mechanism of ATP hydrolysis by beef heart mitochondrial ATPase. Rate constants for elementary steps in catalysis at a single site. *J. Biol. Chem.*, 257(20):12092–12100, October 1982.

[18] Peter Gräber. The H+-ATPase from chloroplasts: Energetics of the catalytic cycle. *Biochim. Biophys. Acta, Bioenerg.*, 1187(2):171–176, August 1994.

[19] O. Panke and B. Rumberg. Kinetic modelling of the proton translocating CF0CF1-ATP synthase from spinach. *FEBS Lett.*, 383:196+, 1996.

[20] Yonatan Savir and Tsvi Tlusty. The Ribosome as an Optimal Decoder: A Lesson in Molecular Recognition. *Cell*, 153(2):471–479, April 2013.
